# Study on the Mechanical Properties and Durability of Recycled Aggregate Concrete under the Internal Curing Condition

**DOI:** 10.3390/ma15175914

**Published:** 2022-08-26

**Authors:** Guanghao Yang, Qiuyi Li, Yuanxin Guo, Haibao Liu, Shidong Zheng, Mingxu Chen

**Affiliations:** 1 School of Civil Engineering, Qingdao University of Technology, Qingdao 266525, China; 2 School of Architectural Engineering, Qingdao Agricultural University, Qingdao 266109, China; 3 Shandong Junhong Environmental Technology Co., Ltd., Zibo 255000, China

**Keywords:** pre-wetted recycled coarse aggregate, internal curing, workability, mechanical properties, durability

## Abstract

Poor mechanical properties and durability of recycled aggregate concrete (RAC) hinder its application in the construction field. In this study, pre-wetted recycled coarse aggregate was used as the internal curing material for prepared RAC with low water-to-binder ratio (W/B), aiming to improve the mechanical properties and durability. The results show that the workability decreases with increasing contents of pre-wetted recycled coarse aggregate. The variation in compressive strength of RAC with different contents of pre-wetted recycled coarse aggregate is obvious within 28 d. After 28 d, the effect of internal curing of pre-wetted recycled coarse aggregate starts to occur, causing a sustained increase in compressive strength. The sealed concrete with 50% and 75% pre-wetted recycled coarse aggregate contents presents the highest compressive strength and better internal curing effect. The pre-wetted recycled coarse aggregate decreases the relative humidity inside the concrete and effectively inhibits the development of shrinkage in the early stages. The RAC with pre-wetted recycled coarse aggregate presents little effect on the drying shrinkage. Additionally, the electric flux of RAC cured for 28 d increases from 561C to 1001C, which presents good resistance to chloride permeation. Microscopic tests indicate that the incorporation of pre-wetted recycled coarse aggregate is beneficial to the improvements of internal structure of RAC.

## 1. Introduction

Recycled aggregate (RA) is prepared by crushing, particle shaping and screening of waste concrete, which was used to replace a certain percentage of natural aggregate (NA) to prepare recycled aggregate concrete (RAC) [1]. The application of RAC can reduce the use of natural aggregate and promote the recycling of waste concrete for the benefit of society and the environment. RA is produced from waste concrete with old cement paste [2]. Many researchers have found that the RA surface is covered with 7% to 30% of old mortar and old cement paste [2,3,4]. Compared with NA, RA presents the characteristic of low apparent density, high porosity and water absorption, leading to inferior mechanical properties and durability of RAC [5,6].

Many studies have been carried out to improve these problems in RAC. Berndt [7] and Mukharjee et al. [8] tried to optimize the microstructure of RA by using volcanic ash reaction of fly ash and silica fume, aiming to improve the interfacial transition zone (ITZ) of RAC. Gao et al. [9] used different nanoparticles to improve the properties of RAC containing recycled clay brick aggregates, finding that the use of nanoparticles improves the mechanical properties and durability of RAC effectively. Tam et al. [10] strengthened the RA based on the mechanism of CO_2_ carbonation, finding that the CaCO_3_ crystals generated by the CO_2_ carbonation reaction filled the internal pores of the RAs and improved the basic properties.

The curing condition of concrete is one of the important factors affecting the mechanical properties and durability [11]. High-performance concrete (HPC) with low water–binder ratio (W/B) cannot achieve the expected curing effect due to the low efficiency of water transfer in the dense structure [12,13]. Therefore, many scholars have also conducted a lot of research on internal curing materials, trying to improve the performances of concrete. Li et al. [14] studied the change in compressive strength of concrete at different ages when SAP dosing was 0~0.6%. It was found that the early compressive strength decreased significantly when a large amount of SAP was incorporated. The strength decreased more significantly when W/B was greater than 0.45. However, the late strength decrease was smaller for concrete with smaller W/B and low SAP admixture. Sun et al. [15] studied the factors affecting the strength of concrete containing SAP, using 50–100 μm particle size SAP with water–cement ratios of 0.37 and 0.3. It was concluded that a lower W/B was more favorable to improve the early strength of concrete and that it was easier to improve the early strength of SAP blended concrete when the SAP particle size was larger. However, the strength at 28 d was negatively correlated. Agostini et al. [16] investigated the mechanical properties of pre-wetted light weight aggregate (LWA) incorporated in low W/B concrete. It was found that the incorporation of pre-wetted LWA reduced the number of CH at a later stage and increased the density of C-S-H gels in the aggregate interface zone. Therefore, the effect of LWA on strength was mainly related to the strength of LWA itself and the effect of pre-wetted LWA internal curing. However, few studies have attempted to use RA as an internal curing material to improve the mechanical properties and durability.

Therefore, in this study, pre-wetted recycled coarse aggregate was used as the internal curing material for prepared RAC with low W/B, aiming to achieve improved mechanical properties and durability of RAC. Recycled coarse aggregate with particle size greater than 4.75 mm was selected and the pre-wetted recycled coarse aggregate was used as an internal curing material. In addition, the effect of pre-wetted recycled coarse aggregate on the properties of concrete were investigated by studying the workability, mechanical properties and durability.

## 2. Materials and Methods

### 2.1. Materials

The test uses P·O 42.5 ordinary Portland cement (OPC) produced by Shandong Shanlv Cement Co., Ltd in Zibo City, China. And Class II fly ash (FA) provided by Shandong Junhong Environmental Protection Technology Co in Zibo City, Shandong Province, China. The chemical composition of both is shown in Table 1. the polycarboxylic acid was used as the water reducing agent. (Shanxi Feike New Material Company in Yuncheng City, China), whose water reduction rate is 35~45%.

**Figure 1:**
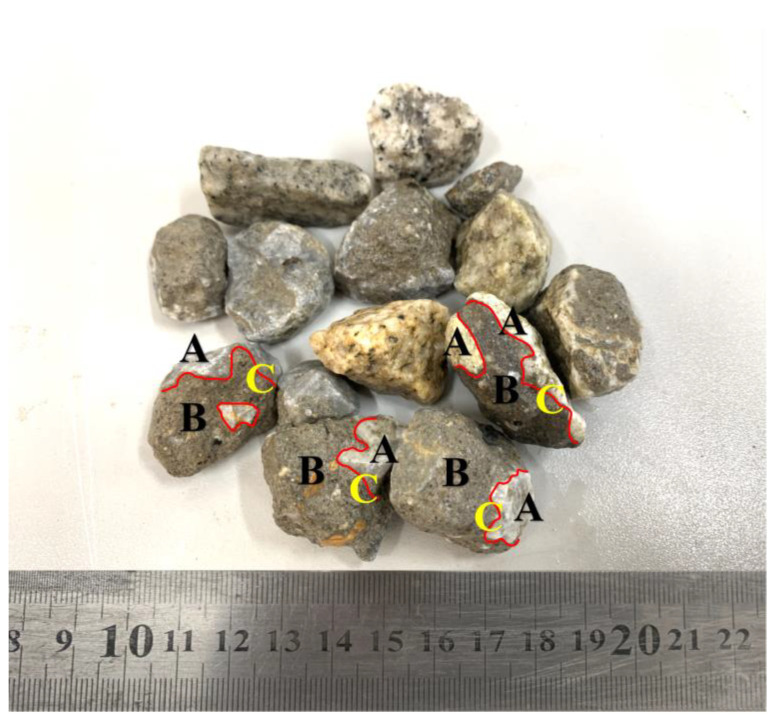
Recycled coarse aggregates consist of (**A**) natural aggregates, (**B**) old mortar, (**C**) interfacial transition zone.

**Figure 2:**
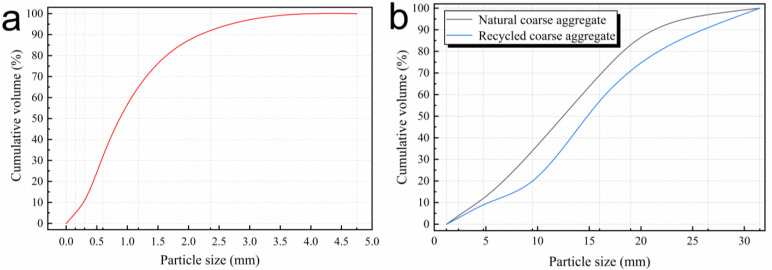
Cumulative distribution of (**a**) natural fine aggregate, (**b**) natural coarse aggregate and recycled coarse aggregate particles.

In order to compare the performance of natural aggregate concrete (NAC) and RAC, natural coarse aggregate and fine aggregate were used. The natural coarse aggregate (NCA) is granite crushed rock produced in Laoshan, whose size is 2.47–19.7 mm. The fine aggregate is Class II river sand produced in Pingdu, Qingdao. The recycled coarse aggregate is obtained from waste concrete through crushing and secondary particle shaping screening, as shown in Figure 1. The recycled coarse aggregate consists of the aggregate surface adhered mortar, and the interface transition zone is the interface of aggregates and cement mortar. Cumulative distribution of aggregate particle size is shown in Figure 2. The basic performance of aggregate is shown in Table 2.

### 2.2. Preparation Procedures

In this study, recycled coarse aggregates with mass fraction replacement rates of 0%, 25%, 50%, 75% and 100% were used instead of NA under two W/B mixed with pre-wetted and dried recycled coarse aggregate, respectively, according to the same mix preparation. The mix proportions are shown in Table 3. The preparation steps are as follows:

(1) OPC, FA, NA, sand and powdered water reducing agent were dry mixed for 5 min in a horizontal concrete mixer.
(2) After the aggregate mixing was completed, mixing water and pre-wetted recycled coarse aggregate were added into the blender and mixed for 5 min.
(3) The fresh concrete was transferred from the blender to the loading container, then shaken fully for 2 min to remove air bubbles and the container was placed in a ventilated area and sealed with cling film.
(4) Concrete was removed from the container after it hardened and treated to be cured under standard conditions.

### 2.3. Testing Methods

#### 2.3.1. Workability

The slump of fresh concrete is tested by the method in the “Standard for test method of performance on ordinary fresh concrete” (GB/T 50080-2016). When the slump is higher than 220 mm, it is required to test the slump expansion.

#### 2.3.2. Mechanical Properties

The mechanical properties of concrete are tested in accordance with the “Standard for test methods of mechanical properties on ordinary concrete” (GB/T 50081-2016). Eighteen cubes (100 mm × 100 mm × 100 mm) for each group of mix ratio are made. After the concrete has been formed and demolded, two types of treatment are carried out, one with foil wrapping and sealing and the other without treatment. The blocks are placed under the same conditions and cured for 7 d, 28 d and 60 d, when they are removed from the surface and tested for compressive strength.

#### 2.3.3. Durability

**Figure 3:**
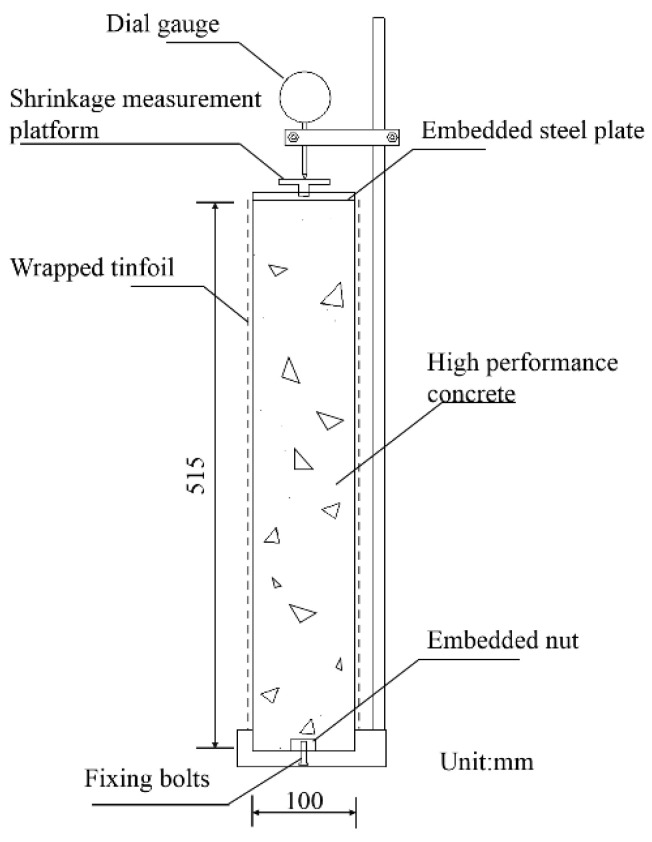
Autogenous shrinkage test rig for concrete.

As shown in Figure 3, the autogenous shrinkage deformation of concrete is tested according to the contact method shrinkage deformation test in the “Standard for test methods of long-term performance and durability of ordinary concrete” (GB/T 50082-2009), and the sensor uses micrometers. The test is carried out on prismatic blocks of size 100 mm × 100 mm × 515 mm. The test is conducted under constant temperature and humidity of (20 ± 2) °C and relative humidity of (60 ± 5)%. Every 12 h, readings are taken to test the autogenous shrinkage values within 7 d. The calculation formula is as in Equation (1).

(1) $$\varepsilon_{\mathrm{st}}=\frac{L_{0}-L_{t}}{L_{b}},$$

where the $\varepsilon_{\mathrm{st}}$ is the test period for t(d) concrete shrinkage rate; the $L_{b}$ is specimen measurement distance (mm); $L_{0}$ is initial value of specimen length (mm);${\;L}_{t}$ is length reading (mm) of the specimen.

**Figure 4:**
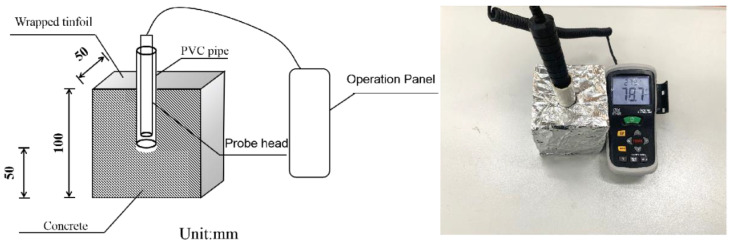
Internal concrete relative humidity test.

In order to study the mechanism of the curing effect inside RA, this study investigates the relative humidity inside the concrete. The test uses concrete specimens with the size of 100 mm × 100 mm × 100 mm, in order to study the humidity distribution law in the center of the concrete inside the specimen at a vertical distance of 5 cm from the surface, PVC pipe with a diameter of 20 mm that intercepts the length of 7 cm pre-buried pipe and a pre-fabricated plastic rod is inserted into the PVC to prevent the concrete from entering the PVC pipe. After the test block is poured, it is wrapped with cling film. As shown in Figure 4, after the same forming time as the self-shrinking test block, the plastic rod is removed and sealed with rubber plugs, the test block is sealed with aluminum foil to prevent water dissipation and placed with the self-shrinking test block under the same constant temperature and humidity conditions for testing and the DT-625 temperature and humidity sensor is used to test the relative humidity inside the concrete. During the test, the probe head is inserted into the PVC pipe, and the value is read when it is placed for 5 min to be stable, and the value is read once every 12 h to test the relative humidity value within 7 d.

In this study, the drying shrinkage is also tested using 100 mm × 100 mm × 515 mm prisms according to the contact method shrinkage deformation test in the “Standard for test methods of long-term performance and durability of ordinary concrete” (GB/T 50082-2009), under constant drying conditions at a temperature of (20 ± 2) °C and relative humidity of (60 ± 5)%. The calculation formula is as in Equation (1).

**Figure 5:**
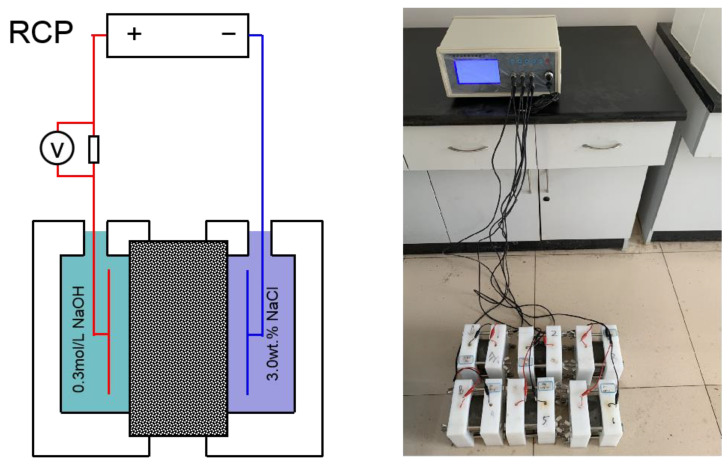
Schematics and test methods of RCP.

In this study, the resistance of concrete to chloride ion attack is tested using the rapid chloride permeability (RCF). Cylindrical specimens of diameter (100 ± 1) mm and height (50 ± 2) mm are used for the test. The specimens are cured under standard conditions for 21 d and then cured by immersion for 7 d. After curing, vacuum saturation is performed to ensure that the specimens contain saturated water. After vacuum satiation, the specimens are sealed in the specimen tank, and NaCl solution with a mass concentration of 3.0% and NaOH solution with a molar concentration of 0.3 mol/L (Figure 5) are added. After connecting the positive and negative electrodes to keep the solution tank full of solution connected to the power supply, the test is conducted under (60 ± 0.1) V, (0~10) A direct current constant power supply, and the electric flux within 6 h is recorded. The electric flux is calculated as Equation (2).

(2) $$Q=900\left(I_{0}+2I_{30}+2I_{60}+\ldots +2I_{t}+\ldots +2I_{300}+2I_{330}+I_{360}\right),$$

where the Q is the total coulomb electric flux (C); the $I_{0}$ is the initial current (A); $I_{t}$ is the current at t minutes (A).

### 2.4. Properties of ITZ

In order to investigate the structure and morphology of the voids near the aggregates and the thickness of the ITZ, the calcium and silicon ratios obtained by SEM and line scanning perpendicular to the ITZ are investigated for the thickness and morphology of the ITZ, respectively.

## 3. Results and Discussion

### 3.1. Workability

**Figure 6:**
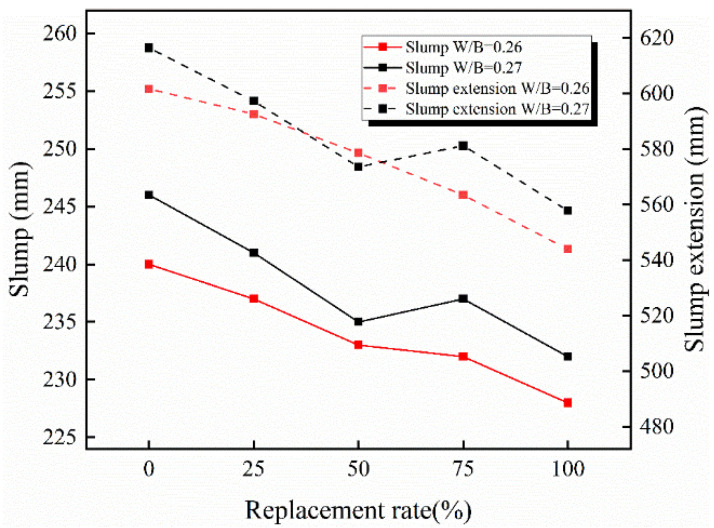
The slump of different concretes.

The slump of RAC with different W/B and replacement ratios of recycled coarse aggregate is shown in Figure 6. The slump of RAC decreases with the increasing replacement ratio of recycled coarse aggregate. Compared with the control group, the slump of RAC with a 100% replacement ratio decreases only 9.62% and 9.51%. The reason is that the low W/B causes the cementitious material to be easily adsorbed on the surface of wetted objects, so a large amount of cementitious material agglomerates around the pre-wetted aggregate when water and pre-wetted recycled coarse aggregate are added. In addition, the friction between recycled coarse aggregates with rough surfaces causes a decrease in concrete slump [17,18]. The addition of recycled coarse aggregate can reduce high water absorption by pre-wetting, so that the water is not reabsorbed during the mixing process. In contrast, the pre-wetted recycled coarse aggregate can effectively improve the workability of concrete. With different W/B, the concrete with higher W/B presents higher slump and better workability. Workability variations in concrete incorporated with pre-wetted recycled coarse aggregate are minimal which could be improved by the addition of admixtures.

### 3.2. Mechanical Properties

#### 3.2.1. Compressive Strength under Normal Conditions

**Figure 7:**
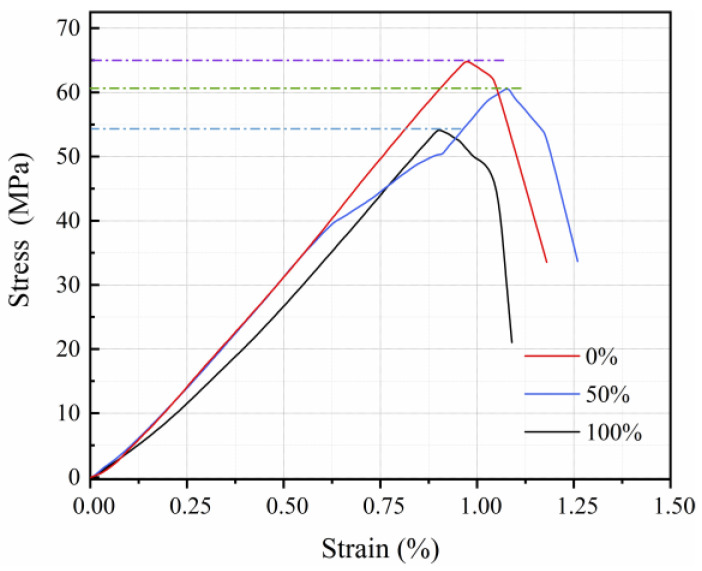
Stress–strain curves of RAC with different pre-wetted recycled coarse aggregate replacement ratios at W/B of 0.26.

As shown in Figure 7 the stress–strain curves of concrete mixed with pre-wetted recycled coarse aggregate have similar development trends, and the full stress–strain curve is divided into two stages: rising and falling. In the early stage of loading, the concrete stress and deformation are linearly related. As the load reaches its peak, a large number of cracks in the concrete produce large displacement, resulting in a rapid decline in bearing capacity. Concrete without recycled coarse aggregate had the highest slope of the stress–strain curve, so it had the highest modulus of elasticity. There was a significant reduction in the strength and modulus of elasticity of RAC after incorporation of pre-wetted recycled coarse aggregates. The pre-wetted recycled coarse aggregate replacement rate of 50% has a higher stress peak compared to the replacement rate of 100%, yet it has a lower modulus of elasticity. It is due to higher pre-wetted recycled coarse aggregate incorporation resulting in amplified aggregate defects, but bringing in more conditioning water ensures higher cement hydration. 

**Figure 8:**
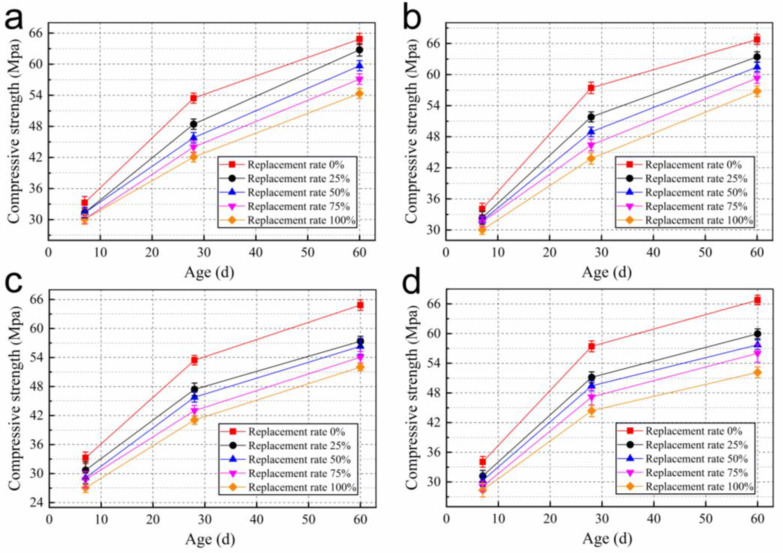
Compressive strength of concrete mixed in pre-wetted RCA with W/B of (**a**) 0.26 and (**b**) 0.27. Compressive strength of concrete mixed in dry RCA with W/B of (**c**) 0.26 and (**d**) 0.27.

The compressive strengths of RAC with different W/B, different ages and replacement rates are shown in Figure 8. As shown in Figure 8a,b, the compressive strength of RAC at each replacement rate is around 30 MPa at 7 d for the same W/B, and decreases with the increase in recycled coarse aggregate replacement rate. However, the cement is not fully hydrated at 7 d, and the cement stone is damaged first under load. Therefore, the effect of mixing recycled coarse aggregate on strength is not obvious [19,20].

After curing for 7 d, the strength of concrete increases with the continuous hydration of cement, and the influence of aggregate and interface on strength is more obvious. With the decrease in relative humidity, pre-wetted recycled coarse aggregate plays a role in internal humidity maintenance. However, due to the low strength of recycled coarse aggregate, the constraint of ITZ on the compressive strength gradually increases [21,22]. The highest growth rate of compressive strength was observed for each group of test specimens up to 28 d, and the growth rate decreased continuously with increasing replacement rate. When mixed with pre-wetted recycled coarse aggregate, the compressive strengths of RAC at 28 d all reached 40 MPa.

**Figure 9:**
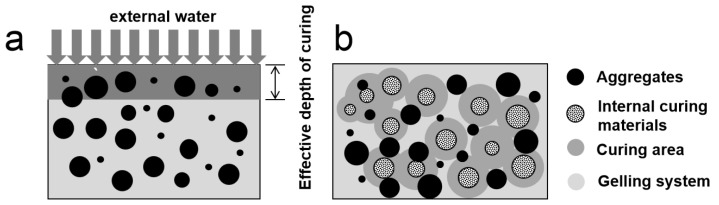
Comparison of (**a**) external and (**b**) internal conservation methods.

After curing for 28 d, the compressive strength difference between the concrete mixed with dry recycled coarse aggregate and the concrete without recycled coarse aggregate was still large. However, the strength growth rate of concrete mixed with pre-wetted recycled coarse aggregate was higher after 28 d. As shown in Figure 8a, the growth rates of compressive strength from 28 d to 60 d reached 22.4%, 25.1%, 27.8% and 29.6%, respectively. It can reach up to twice the strength of concrete without recycled coarse aggregate, and the strength gap between RAC and concrete without recycled coarse aggregate is reduced. Compared with blending into dry recycled coarse aggregate, the strength improvement of RAC can reach up to 9.4% at 60 d after pre-wetting of recycled coarse aggregate. This shows that the effect of pre-wetted recycled coarse aggregate internal curing on the compressive strength of concrete starts to manifest after 28 d. As shown in Figure 9 it is difficult for cement to obtain water from outside due to the dense structure of concrete with low W/B in the later stages [23,24]. As curing continues, the relative humidity inside the concrete decreases as pre-wetted recycled coarse aggregate transfers water through capillary pores. Most of the curing water comes from pre-wetted recycled coarse aggregate, and internal curing becomes the main curing method for RAC.

With the internal curing, the better hydration of cement stone makes up for some of the defects of RAC itself. The difference in compressive strength of RAC with different replacement rates around 60 d is reduced. The 60 d compressive strengths of RAC can all reach more than 50 MPa. 

Strength of concrete is related to the degree of hydration of cement and the strength of aggregates, and the bond strength during ITZ [6]. For the untreated RAC, aggregate defects and ITZ of the recycled coarse aggregate are important factors that affect its strength. Due to the high-level water absorption of recycled coarse aggregate, the dry recycled coarse aggregate will absorb part of the mixing water in the fresh concrete, resulting in a reduction in the effective W/B of the concrete and limiting the hydration of the cement [25]. On the one hand, pre-wetted recycled coarse aggregates effectively improve the degree of hydration of cement around the aggregates [26]. On the other hand, since the cement is attached to the surface of the water-containing material, the cement can be closely adsorbed on the surface of the pre-wetted aggregate and obtain water from it for better hydration in the ITZ. Such factors enable the pre-wetted recycled coarse aggregate internal curing effect to improve the RAC strength.

When W/B is 0.26 and 0.27, the compressive strength of RAC changes similarly. However, the RAC strength at higher W/B is higher in the early stage and the reduction in compressive strength is due to the defects of recycled coarse aggregate. The reason is that more mixing water was provided to hydrate the cement in the early stage. However, after 28 d, the effect of internal curing starts to occur and the effect of W/B on the compressive strength decreases. The difference in compressive strength between these two W/B at 60 d was not significant.

#### 3.2.2. Compressive Strength under Sealed Conditions

**Figure 10:**
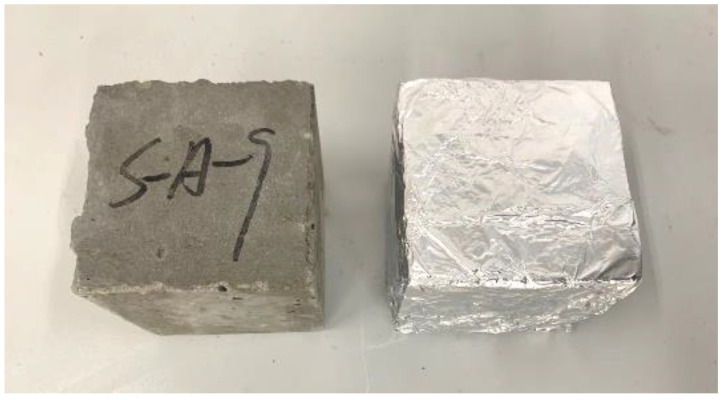
Comparison of test blocks before and after sealing.

The incorporation of pre-wetted recycled coarse aggregate is beneficial to the late development of strength and makes up for some of the strength defects of recycled coarse aggregate itself, but the effect is limited. An appropriate amount of pre-wetted recycled coarse aggregate can not only achieve the best internal curing effect, but also ensure the highest strength in the later stage. Therefore, the concrete specimens just taken out of the molds were sealed and wrapped with tinfoil to bond the joints with glue, and then put into the same conditions as the untreated concrete specimens for curing, as shown in Figure 10.

**Figure 11:**
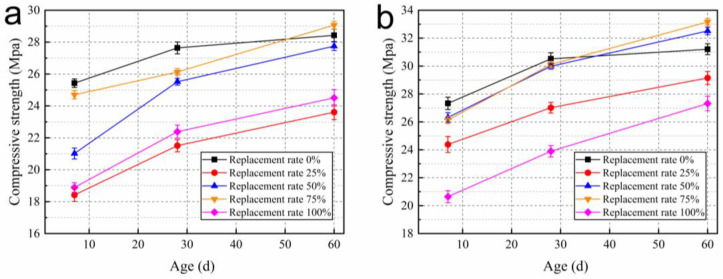
Compressive strength at (**a**) W/B = 0.26, (**b**) W/B = 0.27 under sealed conditions.

Figure 11a,b show the compressive strength of RAC at each age after sealing treatment. It can be seen that the compressive strength of RAC with 100% and 25% replacement rates was the lowest. The compressive strength of the control group was higher until the curing time was 28 d. The RACs with 50% and 75% replacement rates have lower early compressive strengths, but the internal curing effect of the pre-wetted recycled coarse aggregate ensures continuous hydration of the cement. After curing for 28 d, the compressive strength of concrete without recycled coarse aggregate changes less, while the compressive strength of RAC with pre-wetted recycled coarse aggregate increases significantly. The variation in compressive strength under sealed conditions and unsealed conditions is different. The advantage of the internal curing effect under sealed conditions starts to manifest after 7 d as only the internal water of concrete involved in hydration makes the internal relative humidity of concrete decrease faster. The growth rate of the compressive strength shows that the internal curing can effectively improve cement hydration and bond strength of the ITZ, and then increase the compressive strength.

### 3.3. Durability

#### 3.3.1. Autogenous Shrinkage and Relative Humidity

**Figure 12:**
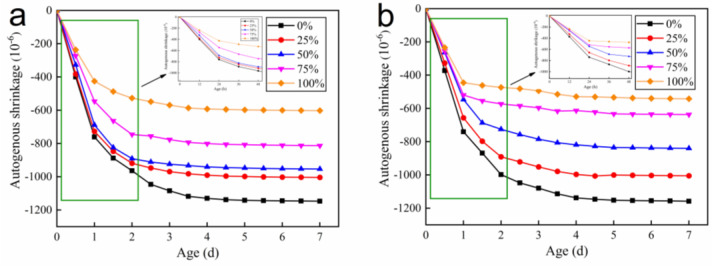

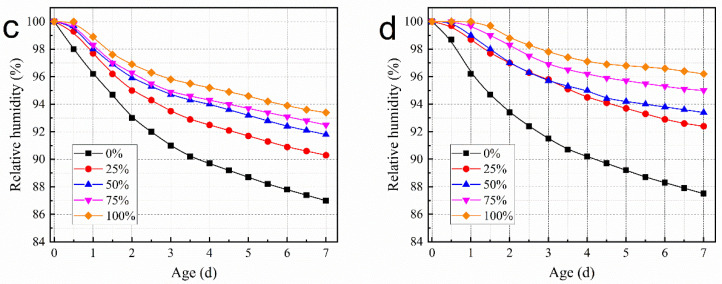
Autogenous shrinkage of concrete with (**a**) W/B = 0.26 and (**b**) W/B = 0.27. Relative humidity of concrete with (**c**) W/B = 0.26 and (**d**) W/B = 0.27.

Concrete with low W/B suffers from large early autogenous shrinkage and easy cracking, which seriously affects the application of concrete [27,28,29]. Incorporation of internal curing materials can effectively reduce the early autogenous shrinkage. In this paper, positive values represent expansion and negative values represent contraction. As shown in Figure 12a,b, the incorporation of pre-wetted recycled coarse aggregate has a significant effect on reducing the concrete autogenous shrinkage. The reason is that concrete with low W/B mixes less water, and as the hydration of cement proceeds the internal relative humidity decreases and the free water content of cement stone capillaries decreases, which in turn generates negative pressure in the capillaries and leads to concrete shrinkage [30,31,32]. In Figure 12a, RAC with 25%, 50%, 75% and 100% replacement rates reduced shrinkage by 6.5%, 9.0%, 23.4% and 45.8% at 2 d compared to the control concrete. The reduction in shrinkage at 2 d reached 27.3% and 42.6%, respectively, which could achieve a shrinkage reduction effect of 75% and 100% for W/B of 0.26 concrete replacement rate. The shrinkage of concrete mixed with pre-wetted recycled coarse aggregate tends to smooth out and shrinkage decreases after 2 d. The control group tends to smooth out after partial shrinkage from 2 d to 7 d. The relative humidity inside the concrete decreases, and the pre-wetted recycled coarse aggregate slowly delivers water to the cement stone capillaries to maintain the stability of relative humidity inside the concrete, thus slowing down the early autogenous shrinkage of concrete.

Figure 12c,d show that the relative humidity in the concrete increases gradually when the content of recycled coarse aggregate increases to 100% and the internal relative humidity of concrete after incorporating pre-wetted recycled coarse aggregate was higher than that of control concrete at the same age. The relative humidity of the concrete was maintained at 90% for 7 d. This indicates that pre-wetted recycled coarse aggregate releases the water absorbed by itself to supplement the water consumed by cement hydration and delays the reduction in internal relative humidity of concrete when the internal relative humidity decreases. Comprehensive analysis of the conservation effect inside the pre-wetted recycled coarse aggregate revealed a high correlation between the trend of RAC autogenous shrinkage and internal relative humidity variation. The effect of pre-wetting recycled coarse aggregates releasing water to maintain relative humidity is the main reason for reduced autogenous shrinkage [33,34]. This shrinkage reduction effect is also more pronounced as the amount of internal curing water introduced increases [35].

For W/B, a higher shrinkage reduction effect of concrete is better, and the reason is adding more mixing water maintains a higher level of relative humidity inside the concrete for the internal curing and better moisturizing effect to ensure the free water content in the cement capillaries [36]. In the process of autogenous shrinkage, the shrinkage of specimens continued to increase in the first 36 h, and the shrinkage of concrete gradually tended to be constant from 36 h to 48 h. After 2 days, the shrinkage was basically stable. This indicates that the setting time of each group of RACs is similar, and the introduction of internal curing water has no effect on the setting time of concrete, so the pre-wetted recycled coarse aggregate will “export” water to the concrete when the concrete cement hydrates, and the internal curing water brought in by recycled coarse aggregate and concrete mixing water can complement each other.

#### 3.3.2. Dry Shrinkage

**Figure 13:**
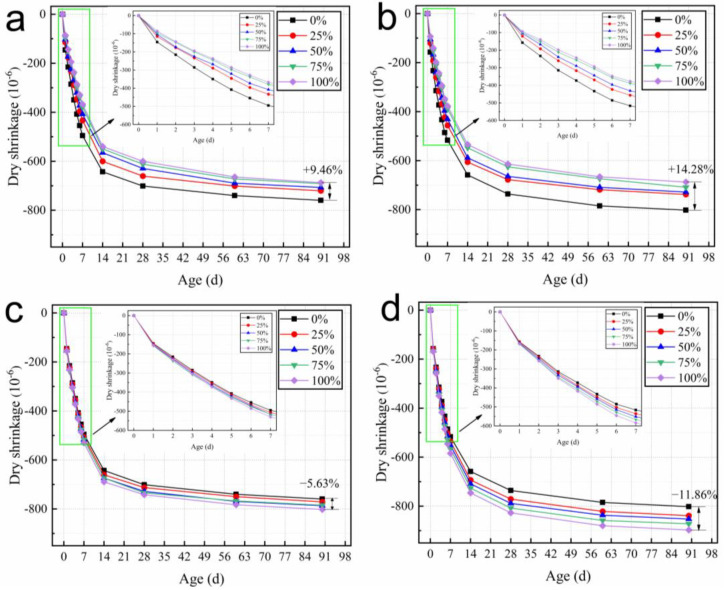
Dry shrinkage of concrete mixed in pre-wetted RCA with W/B of (**a**) 0.26 and (**b**) 0.27. Dry shrinkage of concrete mixed with dry RCA with W/B of (**c**) 0.26 and (**d**) 0.27.

Figure 13 shows the effect of pre-wetted and dried recycled coarse aggregate on the dry shrinkage of concrete in a simulated dry environment. As shown in Figure 13, the trends of dry shrinkage for RACs with different W/B are similar; the dry shrinkage of concrete increases effectively in the first 14 d and changes less after 14 d. However, the mixing of pre-wetted recycled coarse aggregate has a certain shrinkage reduction effect on concrete. Since the fundamental reason for the dry shrinkage is the transport of moisture under the internal and external environment [37,38], the effect of incorporating pre-wetted recycled coarse aggregate on the reduction in concrete shrinkage is not significant. The 90 d concrete with 100% recycled coarse aggregate replacement has only a 9.46% and 14.28% reduction compared to the control concrete. Mixing pre-wetted recycled coarse aggregate can reduce the early dry shrinkage of concrete. Pre-wetted recycled coarse aggregate can release water to ensure stable internal relative humidity during moisture transfer between the early concrete and the outside. The internal relative humidity of the control concrete at the later stage is similar to the external environment, making the moisture transfer less efficient [39], while the internal relative humidity of the concrete incorporated with RAC has a gradient with the external environment leading to a greater rate of change in dry shrinkage at the later stage of RAC.

Considering the mechanism of dry shrinkage, mixing with pre-wetted recycled coarse aggregate can reduce the dry shrinkage of concrete. However, the shrinkage reduction effect of pre-wetted recycled coarse aggregate is not obvious because the final relative humidity reaches the same level as the outside environment. However, the effect of recycled coarse aggregate on the dry shrinkage of concrete can be decreased after pre-wetting treatment compared to the concrete mixed with dry recycled coarse aggregate.

#### 3.3.3. Rapid Chloride Permeability (RCP)

**Figure 14:**
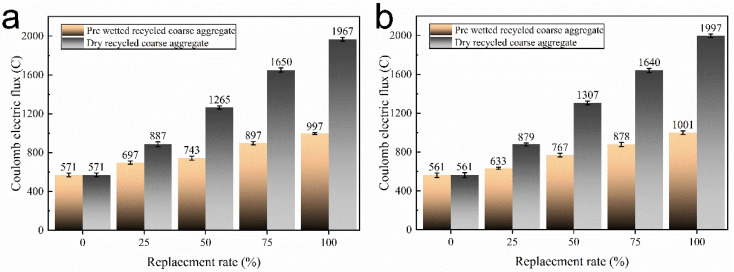
Rapid chloride permeability (RCP) of the concrete with W/B of (**a**) 0.26 and (**b**) 0.27.

Rapid chloride permeability is one of the main indexes to investigate the durability of concrete [40]. Figure 14 demonstrates the chloride ion flux of concrete with different replacement rates of recycled coarse aggregate ratio. As shown in Figure 14, the concrete electric flux increases with the increase in RAC replacement rate. Following pre-wetting treatment of recycled coarse aggregate, the RAC electric fluxes, when cured for 28 d, increase from 561C to 1001C. When W/B is 0.26, compared with the control group for W/B = 0.26, the electric flux increased 126C, 172C, 326C and 426C, respectively, when the replacement rate of pre-wetted recycled coarse aggregate reached 25%, 50%, 75% and 100%, which presents low resistance to chloride ion permeability performance. The reason is that the concrete with low W/B presents a dense surface structure, which can resist the transport of water [41]. 

**Figure 15:**
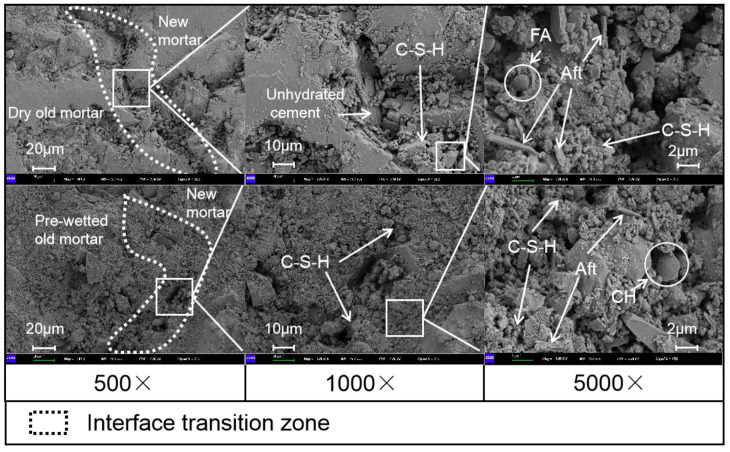
SEM images of ITZ between recycled coarse aggregate old paste and new paste.

Since the high water absorption of recycled coarse aggregate comes from adhering to the old mortar [42], the ITZ microstructure at the adherence of recycled coarse aggregate to the old mortar was analyzed to investigate the reason for the internal curing effect to improve its impermeability. The ITZ microstructure of the pre-wetted and dry recycled coarse aggregate attached to the old mortar with the new mortar in Figure 15 shows the presence of a large number of hydration products around the ITZ, such as hydrated calcium silicate (C-S-H) gels, Ca(OH)_2_ (CH) and rod-shaped ettringite (AFt). The existence of a significant ITZ and cracks in the old mortar of dry recycled coarse aggregate proves that the existence of channels between the old mortar and the new mortar leads to a risk regarding the permeability of the concrete. In contrast, no significant cracks were found between the pre-wetted recycled coarse aggregate old mortar and the new mortar, and the new mortar and the old mortar were tightly connected. Moreover, a large amount of C-S-H gel fills its connecting region in. New gel appears on the surface of pre-wetted old mortar, which may be due to the abundance of water inside the old mortar so that the cement gathers in its pores and the cement hydrates with water to produce a new gel.

In SEM images, ITZ porosity is related to its surface hydration products and porosity decreases with increasingly dense C-S-H gels [43]. A large number of rod-shaped Aft, C-S-H gels and unfilled pores are present near the ITZ of dry old mortar. In contrast, the vicinity of the pre-wetted old mortar ITZ is completely filled with C-S-H gels and almost no pores are visible. The higher porosity of the ITZ enhances the transport of pore solutions [44]. The pre-wetted old mortar can transfer water to the cement paste, making the cement hydration at the ITZ more complete and thus the interface more dense. The presence of a more obvious ITZ and cracks in the dry old mortar may be the main reason for the high degradation of its resistance to chloride ion penetration.

However, compared with NA, recycled coarse aggregate has high porosity, causing a complex ITZ in the RACs [45], which affects the resistance of concrete to chloride ion penetration. Incorporation of pre-wetted recycled coarse aggregate can transfer water to the slurry around the aggregate to ensure continuous cement hydration and improve the effect of a complex ITZ on the chloride ion permeation resistance of concrete.

## 4. Conclusions

According to the performance tests on various aspects of concrete mixed with pre-wetted recycled coarse aggregate, dry recycled coarse aggregate and natural aggregate, as well as the results of the study on different replacements rates of RAC, the conclusions can be drawn as follows:

(1) The workability of concrete decreases with the increase in pre-wetted recycled coarse aggregate replacement rate. However, the difference in workability is small and can be adjusted by admixture addition.
(2) Concrete mixed with pre-wetted RCA showed a greater strength drop in the first 28 d compared to NAC, and the strength gap was gradually reduced after 28 d. Under sealed conditions, the compressive strength of RAC was closest to the compressive strength of control concrete at 50–75% replacement rate. The internal curing effect of higher W/B was more obvious.
(3) Concrete mixed with pre-wetted recycled coarse aggregate delayed the reduction in relative humidity inside the concrete, and the relative humidity inside the concrete mixed with pre-wetted recycled coarse aggregate was guaranteed to be above 90% within 7 d. The autogenous shrinkage reduction effect is obvious.
(4) Mixing pre-wetted recycled coarse aggregate has no significant shrinkage reduction effect on dry shrinkage of concrete in 90 d.
(5) The low W/B concrete has good resistance to chloride ion permeation. The RAC fluxes mixed with pre-wetted recycled coarse aggregate for 28 d curing range from 561C to 1001C, the chloride ion permeability is very low and the resistance to chloride ion permeation is excellent.

## Figures and Tables

**Table 1:** Chemical composition of cement and fly ash.

Materials	CaO (%)	SiO_2_ (%)	Al_2_O_3_ (%)	Fe_2_O_3_ (%)	MgO (%)	Na_2_O (%)	K_2_O (%)
cement	63.75	19.42	6.36	5.42	2.07	0.79	0.56
fly ash	4.81	49.51	31.66	4.23	0.17	1.36	-

**Table 2:** Basic properties of NA and recycled coarse aggregate (RCA).

Properties	Apparent Density (kg/m^3^)	Crush Index (%)	Water Absorption (%)	Pin Flake Content (%)	Stack Density (kg/m^3^)
RCA	2530	11.4	1.6%	2.17	1632
NA	2430	11.1	2.8%	4.15	1518

**Table 3:** Mix proportion of low W/B concrete.

No.	Cement (kg/m^3^)	FA (kg/m^3^)	Sand (kg/m^3^)	NCA (kg/m^3^)	RCA (kg/m^3^)	Water Reducing Agents (%)	Water (kg/m^3^)	W/B
S-26-0	410	135	570	1058	0	1.2	146.9	0.26
S-26-25				794.5	263.5			
S-26-50				529	529			
S-26-75				263.5	794.5			
S-26-100				0	1058			
S-27-0				1058	0		152.6	0.27
S-27-25				794.5	263.5			
S-27-50				529	529			
S-27-75				263.5	794.5			
S-27-100				0	1058			

## Data Availability

Not applicable.

## Abbreviations

The following abbreviations are used in this manuscript:

RA	Recycled Aggregate
NA	Natural Aggregate
RAC	Recycled Aggregate Concrete
ITZ	Interface Transition Zone
OPC	Ordinary Portland Cement (42.5 grade)
W/B	Water-to-Binder Ratio
CH	Ca(OH)_2_
AFt	3CaO·Al_2_O_3_·3CaSO_4_·32H_2_O
C-S-H	xCaO·SiO_2_·yH_2_O
